# New secoiridoid Glycosides from the Roots of *Picrorhiza Scrophulariiflora*

**DOI:** 10.3390/molecules13092049

**Published:** 2008-09-01

**Authors:** Lian-Chun Zou, Tong-Fei Zhu, Hua Xiang, Lu Yu, Zhi-Hui Yan, Shu-Cai Gan, Da-Cheng Wang, Sheng Zeng, Xu-Ming Deng

**Affiliations:** 1 College of Chemistry, Jilin University, Changchun 130062, P.R. China; 2 College of Animal Science and Veterinary Medicine, Jilin University, Changchun 130062, P.R. China; E-mails: xianghuaxh@126.com (Hua Xiang), yulu225@126.com (Lu Yu); 3 Department of Traditional Chinese Materia Medica, Shenyang Pharmaceutical University, Shenyang 110016, P.R. China; 4 Chongqing Medical and Pharmaceutical College, Chongqing 400030, P.R. China

**Keywords:** *Picrorhiza scrophulariiflora*, Secoiridoid glycosides, Picrogentiosides A, B and C, Immunoenhancement

## Abstract

Three new secoiridoid glycosides, named picrogentiosides A (**1**), B (**2**) and C (**3**), have been isolated from the underground parts of *Picrorhiza Scrophulariiflora*, together with the two known compounds plantamajoside (**4**) and plantainoside D (**5**). Their structures were established by spectroscopic analyses and comparisons with data from related compounds. A pilot pharmacological study showed that picrogentiosides A (**1**) and B (**2**) have an immunomodulatory effect *in vitro*.

## Introduction

The plant *Picrorhiza scrophulariiflora* (*Scrophulariaceae*) grows in the high altitude regions (over 4,400 m) in the southeast of Tibet and the northwest of Yunnan in China. The roots of this plant, known to be rich in terpenoids, iridoid glycosides, phenolic glycosides and phenylethanoid glycosides [1,2,3,4], are used in Traditional Chinese Medicine for the treatment of damp-heat dysentery, jaundice and steaming of bone [5]. Many biological activities were reported for its secondary metabolites, including hepatoprotection and immunomodulation activities [4,6].

**Figure 1:**
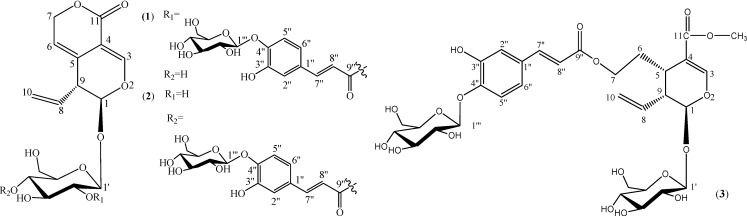
Structures of picrogentiosides A (**1**), B(**2**) and C(**3**).

In our search for immunomodulatory substances from traditional herbs, an aqueous methanol extract from the roots of *P. scrophulariiflora* yielded three new caffeoyl glycosides [7]. Here we report the isolation and characterization of three new secoiridoid glycosides, named picrogentiosides A (**1**), B (**2**) and C (**3**) (Figure 1), as well as the two known compounds plantamajoside (**4**) and plantainoside D (**5**). Results of an *in vitro* pilot pharmacological study on the immunomodulatory effect of picrogentiosides A-C vitro are also reported to provide a reference for further studies.

## Results and Discussion

The *n*-BuOH fraction from the 90% EtOH ext of the roots of *Picrorhiza Scrophulariiflora* was passed over a macroporous D-101 resin column and eluted with successive water and ethanol washes (water and 30%, 50% and 100% ethanol). The 50% EtOH fraction was passed over a silica gel column, using mixts. of CHCl_3_-MeOH of increasing polarity to give eight fractions (Fr. 1-Fr. 8). After further purification by silica gel, Sephadex LH-20 and repeated reversed-phase HPLC using MeOH/H2O, Fr. 3 yielded three new secoiridoid glycosides, picrogentiosides A(**1**), B(**2**) and C(**3**), and the two known compounds plantamajoside (**4**) [8] and plantainoside D (**5**) [8]. Their structures were elucidated by extensive spectroscopic and chemical analyses.

Picrogentioside A (**1**) was obtained as a yellow amorphous powder, with [*α*]^25^_D_-153.7° (*c* 0.49, MeOH) and its molecular formula was determined as C_31_H_36_O_17_ by HR-ESI-MS (*m/z* 703.1859; calculated for C_31_H_36_O_17_Na 703.1850). UV (215, 277 nm) and IR (3437, 1712, 1645, 1606, 1510) absorptions suggested the presence of hydroxyl, *α*, *β*-unsaturated ester and aromatic groups. 

The ^1^H NMR spectrum of **1** (Table 1) showed the presence of an acetal proton [*δ*_H_ 5.44 (1H, d, *J* = 3.2 Hz), H-1], an oxygenated methylene proton [*δ*_H_ 5.02 (1H, m), 5.07 (1H, m), H_2_-7], two olefinic protons [*δ*_H_ 7.28 (1H, br. s), H-3; 5.60 (1H, m), H-6], one methine proton [*δ*_H_ 3.40, (1H, m), H-9], and one terminal vinyl group [*δ*_H_ 5.13 (1H, dd, *J* = 2.0, 10.4 Hz), 5.20 (1H, dd, *J* = 2.0, 16.8 Hz), H_2_-10; 5.68 (1H, ddd, *J* = 6.8, 10.4, 16.8Hz), H-8], due to one gentiopicroside moiety [9]. Furthermore, the spectrum showed the presence of a (*trans*)-caffeoyl moiety [7,10], which was evident from three aromatic H-atoms forming an ABX system [*δ*(A) 7.12, *δ*(B) 7.09, *δ*(X) 7.03; *J* (AB) = *J* (AX) ≈ 0, *J* (BX) = 8.0 Hz], and two H-atoms of an (*trans*)-configured C=C bond at *δ*(H) 6.18, 7.44 (2d, *J* = 16.0 Hz each).

In addition, two anomeric H-atoms were observed at *δ*_H_ 4.75 (1H, d, *J* = 8.0 Hz) and *δ*_H_ 4.78 (1H, d, *J* = 7.2 Hz), which combined with the ^13^C-NMR signals at *δ*c 101.6-60.7 (see Table 1), are typical for two *β*-glucopyranosyl moieties. The sugar component was identified as d-glucopyranoside by co-HPLC analysis of its 1-[(*S*)-*N*-acetyl-α-methylbenzylamino]-1-deoxyalditol acetate derivative with the same derivative of the standard sugar [11]. The relatively large *J* value of the two anomeric protons indicated that the glucoside linkage had a β configuration.

**Table 1:** ^13^C-(100 MHz) and ^1^H-(400 MHz) NMR Data of **1** and **2** (DMSO-*d*_6_)^a^.

position	picrogentioside A (1)		picrogentioside B (2)		picrogentioside C (3)	
	C	H (*J*, Hz)	C	H (*J*, Hz)	C	H (*J*, Hz)
secoiridoid						
1	95.9	5.44, d, (3.2)	95.5	5.60, d, (3.2)	95.6	5.50, d, (6.4)
3	150.8	7.28, br.s	151.4	7.40, br.s	152.0	7.49, s
4	103.7		103.4		109.5	
5	124.7		124.9		30.0	2.80, dd, (5.6,12.0)
6	116.1	5.60, m	116.3	5.65, m	28.9	1.77, dd, (6.8, 13.6),1.94, dd, (6.8, 13.6)
7	68.7	5.02, m5.07, m	69.1	4.98, m5.05, m	62.8	4.16, m4.24, m
8	133.7	5.68, ddd, (6.8, 10.4, 16.8)	134.0	5.71, ddd, (6.8, 10.4, 16.8)	134.5	5.73, ddd, (8.4, 10.0, 18.0)
9	44.0	3.40, m	44.3	3.41, m	43.1	2.60, dd, (6.4, 13.2)
10	117.7	5.13, dd, (2.0, 10.4);5.20, dd, (2.0, 16.8)	118.0	5.19, dd, (2.0, 10.4);5.24, dd, (2.0, 16.8)	119.0	5.25, br.d, (11.2),5.27, br.d, (17.2)
11	162.2		162.7		166.6	
CH_3_O					51.0	3.61, s
caffeoyl						
1''	128.7		128.7		128.6	
2''	115.0	7.12, br.s	114.9	7.15, br.s	114.9	7.13, br. s
3''	146.7		146.9		146.9	
4''	147.4		147.4		147.4	
5''	115.8	7.09, br.d, (8.0)	116.1	7.11, br. d, (8.0)	116.1	7.11, br. d, (8.4)
6''	120.7	7.03, d, (8.0)	120.6	7.11, br. d, (8.0)	120.8	7.11, br. d, (8.4)
7''	144.6	7.44, d, (16.0)	144.1	7.51, d, (16.0)	144.4	7.50, d, (16.0)
8''	116.1	6.18, d, (16.0)	115.9	6.40, d, (16.0)	115.9	6.34, d, (16.0)
9''	165.1		165.9		166.2	
Glc-1						
1'	95.7	4.78, d, (7.2)	98.1	4.66, d, (7.6)	98.7	4.53, d, (8.0)
2'	73.0	4.60, dd, (8.4, 7.2)	72.5	3.17-3.49, m﹡	76.7	3.15, m
3'	73.7	3.15-3.51, m﹡	73.2	3.17-3.49, m﹡	73.0	2.97, t, (8.0)
4'	70.1	3.15-3.51, m﹡	71.2	4.61, m	70.0	3.03, t, (9.2)
5'	77.5	3.15-3.51, m﹡	75.8	3.17-3.49, m﹡	77.3	3.30, m
6'	60.8	3.47, m; 3.71, m	60.7	3.49, m﹡; 3.70, m	61.1	3.43, m; 3.68, m
Glc-2						
1'''	101.6	4.75, d, (8.0)	101.6	4.77, d, (7.2)	101.6	4.78, d, (7.2)
2'''	75.8	3.15-3.48, m﹡	75.8	3.17-3.49, m﹡	75.8	3.28, m
3'''	73.2	3.15-3.48, m﹡	73.2	3.17-3.49, m﹡	73.2	4.29, m
4'''	69.7	3.15-3.48, m﹡	69.8	3.17-3.49, m﹡	69.8	3.15, m
5'''	77.2	3.15-3.48, m﹡	77.2	3.17-3.49, m﹡	77.2	3.31, m
6'''	60.7	3.48, m﹡; 3.72, m	60.7	3.48, m﹡; 3.70, m	60.7	3.43, m; 3.68,m

^a^ Chemical shift (*δ*) given in ppm ﹡ signal pattern unclear due to overlapping.

**Figure 2:**
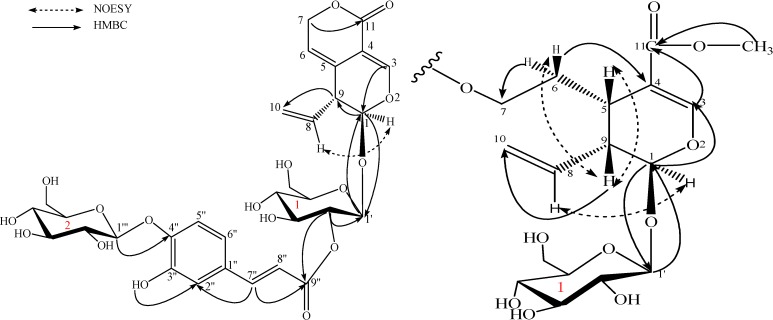
Important HMBC (H→C) and NOESY correlations for picrogentioside A (**1**) and picrogentiosides C (**3**).

The detailed analysis of ^1^H-NMR, TCOSY, ^13^C-NMR, HMQC and HMBC spectra of **1** fixed the connections between the various moieties. The HMBC (Figure 2) correlation between H-1''' (*δ*_H_ 4.75) and C-4''(*δ*c 147.4) demonstrated that glucose 2 was connected to the C-4'' oxygen atom. The downfield shift of the oxygenated methenyl proton at *δ*_H_ 4.60 (H-2') suggested that the caffeoyl moiety was attached at C-2' of the glucose 1. This was confirmed by HMBC correlation between H-2' (*δ*_H_ 4.60) and the C-1' (*δ*c 95.7), and the correlation between H-2' (*δ*_H_ 4.60) and the carbonyl carbon C-9″ (*δ*c 165.1). The relative stereochemistry of **1** was determined from the NOESY spectrum. Correlation between H-5 (*δ*_H_ 5.44) and H-8 (*δ*_H_ 5.68) showed that the two protons are positioned on the same side. Thus, the structure of **1** were determined as shown in Figure 1.

Picrogentioside B (**2**), a yellow amorphous powder, showed [*α*]^25^_D_-144.3° (*c* 0.35, MeOH). The HR-ESI-MS analysis established its molecular formula as C_31_H_36_O_17_. The UV, IR, ^1^H-NMR and the ^13^C-NMR spectral features of **2** were quite similar to those of **1** (Table 1), suggesting the presence of gentiopicroside, caffeoyl and glucose moieties. The difference between **2** and **1** was traced to differences in the glycosidic linkage between the caffeoyl moiety and glucopyranosyl moiety 1. Comparison of the NMR spectra of **2** and **1** also showed a conspicuous deshielding of the oxygenated methenyl proton at *δ*_H_ 4.61 (H-4'), revealing that the caffeoyl moiety should be linked to C-4' of the glucoside 1, as indicated by the HMBC crosspeak between this carbon (*δ*_C_ 165.9) and the anomeric proton (*δ*_H_ 4.61). Acid hydrolysis of **2** gave only d-glucose. Accordingly, the structure of **2** was elucidated to be as shown in Figure 1.

Picrogentioside C (**3**) a yellow amorphous powder, [*α*]^25^_D_-208.3°(*c* 0.53, MeOH). The molecular formula C_32_H_42_O_18_ was assigned for **3** on the basis of HR-ESIMS at *m/z* [M+Na]^+^ (calculated for C_32_H_42_O_18_Na 737.2269). The NMR spectroscopic data of **3** were similar to those of **1** (Table 1), suggesting a close relationship. Structural assessment of **3** was accomplished using a combination of NMR techniques, along with comparisons to the assignments of **1** and **2**.

The ^1^H-NMR spectrum of **3** showed the presence of a methoxyl proton [*δ*_H_ 3.61(3H, s), CH_3_O], an acetal proton [*δ*_H_ 5.50 (1H, d, *J* = 6.4Hz), H-1], an oxygenated methylene proton [*δ*_H_ 4.16 (1H, m), 4.24 (1H, m), H_2_-7], an olefinic proton [*δ*_H_ 7.49, s, H-3], a methylene proton [*δ*_H_ 1.77 (1H, dd, *J* = 6.8, 13.6 Hz), 1.94 (1H, dd, *J* = 6.8, 13.6Hz), H_2_-6], two methine protons [*δ*_H_ 2.80 (1H, dd, *J* = 5.6, 12.0 Hz), H-5; 2.60 (1H, dd, *J* = 6.4, 13.2 Hz), H-9], and one terminal vinyl group [*δ*_H_ 5.25 (1H, br.d, *J* = 11.2 Hz), 5.27 (1H, br.d, *J* = 17.2 Hz), H_2_-10; 5.73 (1H, ddd, *J* = 8.4, 10.0, 18.0 Hz), H-8]. These signals indicated one secologanol moiety [12,13,14]. Furthermore, the presence of *β*-d-glucopyranosyl and (*trans*)-caffeoyl moieties and the connection between caffeoyl moiet and glucose 2 were confirmed using the same methods as for the structure elucidation of **1**. As compared with the secologanol moiety [12,13,14] signals, the caffeoyl moiety should be attached at C-7 of the secologanol moiety by the downfield shift of the C-7 protons at *δ*_H_ 4.16 and 4.24 (H_2_-7) and of the C-7 signal *δ*c 62.8. 

The relative configuration of **3** was determined by analysis of NMR coupling constants and with the aid of NOESY experiments. A *J* (1, 9) value of 6.4 Hz suggested a trans-diaxial orientation for H-1 and H-9 [15,16,17], and NOESY cross-peaks between H-5 and H-9, as well as between H*_β_*-6 and H-9, revealed that they were on the same side of the molecular plane (*β*). From the above data, the structure of **3** was established as shown.

### Biological activity

**Figure 3:**
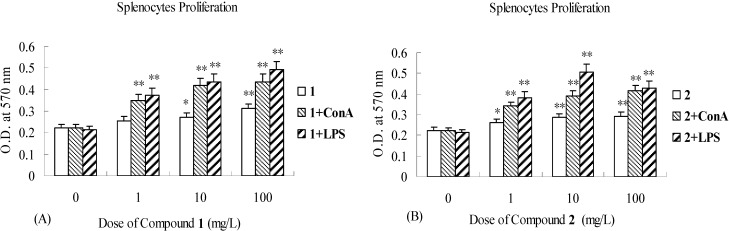
Compounds **1** and **2** enhanced the proliferation of splenocytes *in vitro*. The results were from three independent experiments and present as mean ± SD. * represent p<0.05 and ** represent P<0.01.

Compounds **1** and **2** enhanced proliferations of splenocytes *in vitro* as shown in Figure 3, while **3** statistically had no enhancing effect (data not shown). Many immune processes require a balance between apoptosis and proliferation of splenocytes. All somatic cells proliferate via a mitotic process as determined by progression through the cell cycle. LPS and Con A are well-known lymphocyte mitogens. Con A acts directly on T cells while LPS acts on B cells. The assessment of substances that either promote or inhibit cell proliferation is crucial to the study of cell biology and drug discovery. In our study, **1** and **2** could enhance splenocyte proliferation significantly compared to controls with/without mitogen while **3** could not. Our results suggest that **1** and **2** could enhance immunocyte function, both cellular immunity and humoral immunity, implying that **1 ** and **2** might have a beneficial effect on regulating immune functions while **3** would not [18,19]. The molecular target(s) of **1** and **2** and the potential immunomodulatory effects of **1** and **2** *in vivo* require further study. 

## Experimental

### General

UV spectra were recorded on a Milton Roy Spectronic 1201 spectrophotometer and FTIR spectra were measured on a Perkin-Elmer 157G infrared spectrophotometer. Optical rotations were obtained using a Perkin-Elmer 241-MC polarimeter. ^1^H-NMR (400 MHz) and ^13^C-NMR (100 MHz) spectra were obtained on a Bruker AV400 spectrometer with DMSO-*d*_6_ as the solvent and TMS as an internal standard. HR-ESI-MS data were measured with a Bruker AOEXIII 7.0 TESLA FTMS. HPLC was conducted using reverse phase columns (mighty sil RP-18 and 8, Kantho Chemical Co. Ltd) with the MeOH-H_2_O solvent system. Colum chromatography was carried out on silica gel (Qingdao Marine Chemical Company China, 200-300 mesh) and Sephadex LH-20 (Amersham Pharmacia Biotech AB). Silica GF254 for TLC was produced by Qingdao Marine Chemical Company China and Merck Company. All chemicals used were of biochemical reagent grade. Lipopolysaccharide (LPS), concanavalin A (Con A) and MTT were produced by Sigma (USA). The microplate reader was made by TECAN (Austria).

### Plant Material

Roots of *Picrorhiza scrophulariiflora* were collected in October 2006 in Sichuan Province, P.R. China and identified by Prof. Qi-shi Sun (Shenyang Pharmaceutical University). A voucher specimen has been deposited in the Herbarium of School of Traditional Chinese Medicines of Shenyang Pharmaceutical University, China.

### Extraction and Isolation

The dried and ground roots (underground parts) of *Picrorhiza scrophulariiflora* (3.0 kg) were successively extracted three times with 90% EtOH (3 L each time) under reflux. After removal of the solvent *in vacuo*, the residue (1.6 kg) was suspended in H_2_O (5 L) and then extracted successively with petroleum ether (b.p. 60-90°C), EtOAc and *n*-BuOH. The *n*-BuOH layer was concentrated *in vacuo* to give a viscous residue (500 g), which was then dissolved in water (2 L) and subjected to macroporous D-101 resin column chromatography and eluted successively with water and ethanol (water, 30%, 50% and 100% ethanol). The 50% EtOH eluted fraction was evaporated *in vacuo* to yield a residue (140 g) that was subjected to silica gel column chromatography eluting with mixtures of CHCl_3_-MeOH of increasing polarity (1:0 ~ 0:1) to give eight fractions (Fr.1-8). Fraction 3 (28.5 g) was chromatographed over silica gel using a EtOAc-MeOH (1:0 ~ 0:1) gradient system as the eluent to yield five fractions: 3A-3E. Fraction 3A (5.8 g) was purified by Sephadex LH-20 chromatography (MeOH) and further separated by reversed-phase HPLC using MeOH-H_2_O (48:52/46:54) as the mobile phase to yield compounds **1** (25.8 mg), **2** (23.6mg), **3** (17.4mg), **4** (10.3mg) and **5** (13.3 mg).

*Picrogentioside A* (**1**): Yellow amorphous powder; [*α*]^25^_D_-153.7° (*c* 0.49, MeOH); UV(MeOH) *λ*_max_ nm (log*ε*): 215 (3.20), 277 (3.11); IR (KBr) *ν*_max_cm^-1^: 3522, 3437, 3297, 2899, 2257, 1712, 1645, 1606, 1510, 1403; for ^1^H- and ^13^C-NMR spectra, see Table 1; HR-ESI-MS *m/z* 703.1859 (calculated for C_31_H_36_O_17_Na 703.1850).

*Picrogentioside B* (**2**): Yellow amorphous powder; [*α*]^25^_D_-144.3° (*c* 0.35, MeOH); UV (MeOH) *λ*_max_ nm (log*ε*): 208 (3.41), 271 (3.28); IR (KBr) *ν*_max_cm^-1^: 3525, 3436, 3305, 2897, 2257, 1701, 1622, 1609, 1511,1408; for ^1^H- and ^13^C-NMR spectra, see Table 1; HR-ESI-MS *m/z* 703.1857 (calculated for C_31_H_36_O_17_Na 703.1851).

*Picrogentioside C* (**3**): Yellow amorphous powder; [*α*]_D_-208.3°(*c* 0.53, MeOH); UV (MeOH) *λ*_max_ nm (logε): 209 (3.37), 287 (3.15); IR (KBr) *ν*_max_cm^-1^: 3522, 3436, 3306, 2889, 2263, 1703, 1629, 1617, 1512, 1433, 1271, 1216, 1169, 966, 904, 835, 753, 711; for ^1^H- and ^13^C-NMR spectra, see Table 1; HR-ESI-MS *m/z* 737.2249 (calculated for C_32_H_42_O_18_Na 737.2269).

*Plantamajoside* (**4**): White amorphous powder; ^1^H-NMR *δ*: 7.44 (1H, d, *J* = 16.0Hz, H-7′), 6.27 (1H, d, *J* = 16.0Hz, H-8′), 7.03 (1H, br.s, H-2′), 6.99 (1H, br. D., *J* = 8.0 Hz, H-6′), 6.75 (1H, d, *J* = 8.0 Hz, H-5′), 6.63 (1H, d, *J* = 2.0 Hz, H-2), 6.62 (1H, d, *J* = 8.0 Hz, H-5), 6.49 (1H, dd, *J* = 8.0, 2.0 Hz, H-6), 3.61 (1H, m, H-8), 3.88 (1H, m, H-8), 2.91 (2H, t, *J* = 8.4 Hz, H-7), 4.38 (1H, d, *J* = 8.4 Hz, H-1′′), 4.36 (1H, d, *J* = 8.4 Hz, H-1′′′); ^13^C-NMR *δ*: 129.1 (C-1), 116.3 (C-2), 144.9 (C-3), 143.5 (C-4), 115.4 (C-5), 121.1 (C-6), 35.0 (C-7), 70.1 (C-8), 125.6 (C-1′), 114.8 (C-2′), 145.5 (C-3′), 148.3 (C-4′), 115.7 (C-5′), 119.5 (C-6′), 114.2 (C-7′), 144.9 (C-8′), 165.7 (C-9′), 102.1 (C-1′′), 73.2 (C-2′′), 82.9 (C-3′′), 69.4 (C-4′′), 74.5 (C-5′′), 60.7 (C-6′′), 104.5 (C-1′′′), 74.4 (C-2′′′), 76.2 (C-3′′′), 69.7 (C-4′′′), 76.7 (C-5′′′), 60.7 (C-6′′′)

*Plantainoside D* (**5**): White amorphous powder; ^1^H-NMR *δ*: 7.47 (1H, d, *J* = 16.0 Hz, H-7′), 6.29 (1H, d, *J* = 16.0 Hz, H-8′), 7.05 (1H, d, *J* = 1.6 Hz, H-2′), 6.96 (1H, dd, *J* = 8.0, 1.6 Hz, H-6′), 6.76 (1H, d, *J* = 8.0 Hz, H-5′), 6.61 (1H, d, *J* = 2.0 Hz, H-2), 6.58 (1H, d, *J* = 8.0 Hz, H-5), 6.45 (1H, dd, *J* = 8.0, 2.0 Hz, H-6), 3.61 (1H, dd, *J* = 7.6, 17.2 Hz, H-8), 3.80 (1H, dd, *J* = 7.6, 17.2 Hz H-8), 2.67 (2H, t, *J* = 7.6 Hz, H-7), 4.37 (1H, d, *J* = 8.0 Hz, H-1′′), 4.34 (1H, d, *J* = 7.6 Hz, H-1′′′); ^13^C NMR *δ*: 129.3 (C-1), 116.3 (C-2), 145.0 (C-3), 143.5 (C-4), 115.5 (C-5), 121.5 (C-6), 35.1 (C-7), 70.2 (C-8), 125.4 (C-1′), 114.9 (C-2′), 145.6 (C-3′), 148.6 (C-4′), 115.8 (C-5′), 119.5 (C-6′), 113.7 (C-7′), 145.4 (C-8′), 166.5 (C-9′), 102.2 (C-1′′), 73.2 (C-2′′), 87.6 (C-3′′), 68.6 (C-4′′), 73.9 (C-5′′), 63.3 (C-6′′), 104.1 (C-1′′′), 72.1 (C-2′′′), 76.1 (C-3′′′), 70.2 (C-4′′′), 76.9 (C-5′′′), 61.1 (C-6′′′)

### Acid hydrolysis of **1**, **2** and **3**

A solution of each compound (**1**, **2** and **3**) (6 mg) in 2 N TFA (3 mL) was refluxed at 100°C for 3 h. The reaction mixture was extracted with EtOAc. The EtOAc extract of **1**, **2** and **3** was proven to contain caffeic acid by direct TLC comparison with authentic samples. d-glucose was found as the only sugar present in the water part following the procedure of Oshima, Yamauchi and Kumanotani [12]. The H_2_O layer was neutralized by passing through an ionexchange resin (Amberlite MB-3) column and concentrated under reduced pressure to dryness. The residue was dissolved in H_2_O (1 mL), to which L-(-)-α-methylbenzylamine (5 mg) and NaBH_3_CN (8 mg) in EtOH (1mL) were added. The mixture was stirred at 40 °C for 4 h, then acidified by addition of glacial HOAc (0.2 mL) and evaporated to dryness. The resulting solid was acetylated with Ac_2_O (0.3 mL) in pyridine (0.3 mL) for 24 h at room temperature. After evaporation, H_2_O (1 ml) was added to the residue. The solution was passed through a Sep-Pak C_18_ cartridge and washed with H_2_O and CH_3_CN. The CH_3_CN eluate was analyzed by HPLC under the following conditions: solvent, MeCN-H_2_O (2:3); flow rate, 0.8 mL/min; detection, UV 230 nm. The derivative of d-glucopyranose was detected with the *t*_R_ of 17.3 min.

### Immunomodulatory effects study of **1**, **2** and **3**

The compounds **1**, **2** and **3** (terminal concentrations of 1, 10 and 100 mg/L each) were added to the culture of splenocytes of mice alone in control and combined with either Con A at a terminal concentration of 5 mg/L or LPS at 20 mg/L for 48 h. Cell proliferation was measured using the MTT assay [18,19]. 
